# Growing magma chambers control the distribution of small-scale flood basalts

**DOI:** 10.1038/srep16824

**Published:** 2015-11-19

**Authors:** Xun Yu, Li-Hui Chen, Gang Zeng

**Affiliations:** 1 State Key Laboratory for Mineral Deposits Research, School of Earth Sciences and Engineering, Nanjing University, Nanjing 210023, China

## Abstract

Small-scale continental flood basalts are a global phenomenon characterized by regular spatio-temporal distributions. However, no genetic mechanism has been proposed to explain the visible but overlooked distribution patterns of these continental basaltic volcanism. Here we present a case study from eastern China, combining major and trace element analyses with Ar–Ar and K–Ar dating to show that the spatio-temporal distribution of small-scale flood basalts is controlled by the growth of long-lived magma chambers. Evolved basalts (SiO_2_ > 47.5 wt.%) from Xinchang–Shengzhou, a small-scale Cenozoic flood basalt field in Zhejiang province, eastern China, show a northward younging trend over the period 9.4–3.0 Ma. With northward migration, the magmas evolved only slightly ((Na_2_O + K_2_O)/MgO = 0.40–0.66; TiO_2_/MgO = 0.23–0.35) during about 6 Myr (9.4–3.3 Ma). When the flood basalts reached the northern end of the province, the magmas evolved rapidly (3.3–3.0 Ma) through a broad range of compositions ((Na_2_O + K_2_O)/MgO = 0.60–1.28; TiO_2_/MgO = 0.30–0.57). The distribution and two-stage compositional evolution of the migrating flood basalts record continuous magma replenishment that buffered against magmatic evolution and induced magma chamber growth. Our results demonstrate that the magma replenishment–magma chamber growth model explains the spatio-temporal distribution of small-scale flood basalts.

Large igneous provinces (LIPs) and monogenetic volcanic fields are the two end-member surface expressions of intra-continental basaltic magmatism. LIPs are characterized by prodigious volumes of tholeiitic basalts erupted over a short time, usually <1 Myr^1^,^2^, whereas monogenetic volcanic fields are characterized by small eruptive volumes issued from individual vents, each with only a single eruptive episode^3^. However, many other intra-continental basaltic fields span hundreds to thousands of square kilometers and hundreds to thousands of meters in elevation, showing little connection with mantle plume or hotspot processes; examples include the Miocene Vogelsberg basalts of the central European volcanic province^4^, Pliocene basalts of the Newer Volcanics Province in southeastern Australia^5^, late Cenozoic intra-plate basaltic fields of the western United States^6^, and dozens of late Cenozoic small-volume flood basalt fields in eastern China^7^. In addition to their moderate eruptive volumes (<10^^5^ km^3^), these basalts are mainly tholeiitic, and erupted with a regular spatio-temporal distribution over several million years (e.g., the Chifeng flood basalts^8^,^9^), and cannot be classified as either of the two end-members of intra-continental basaltic volcanism. Here, we refer to such volcanic fields as ‘small-scale continental flood basalts’.

The distribution of basaltic volcanism can reflect melting mechanisms in the source, magma evolution in the magma chamber and/or during ascent, and the eruption pattern of the magma. Previous works have suggested that the movement and structure of mantle plumes, as well as the rupturing of subducting slabs, can explain the distribution of LIPs^10^,^11^, while regional tectonic stresses control the distribution of monogenetic volcanic fields^12^,^13^. However, little is known about the mechanism resulting in the regular distribution of small-scale continental flood basalts. Here, we present a case study of a small flood basalt field from eastern China to determine the role of the magma chamber in the formation and distribution of eruptive centers within small-scale continental flood basalts.

## Geologic setting

**Figure 1:**
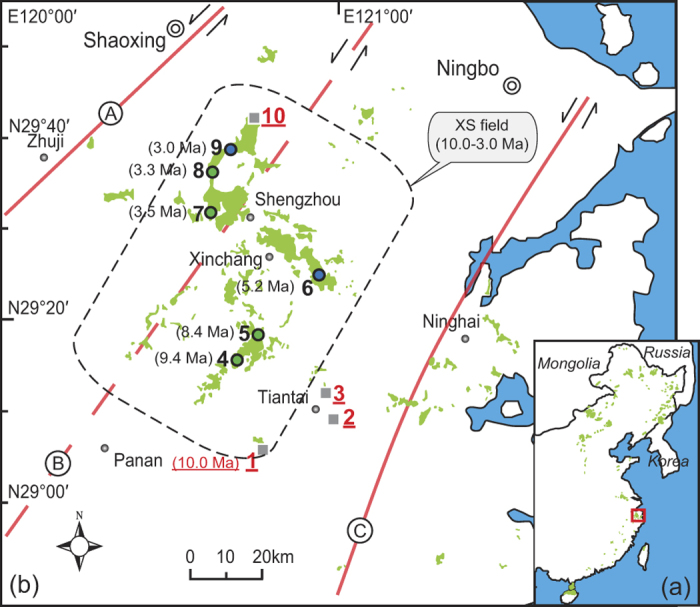
Simplified geologic maps for distribution of Cenozoic basalts in eastern China (**a**) and Zhejiang province (**b**). Three major wrench faults in Zhejiang province include Jiangshan-Shaoxing fault (**A**), Lishui-Yuyao fault (**B**), and Zhenhai-Wenzhou fault (**C**). The sampling locations from south to north: 1-Jiuliping, 2-Dongheng, 3-Guoqingsi, 4-Hanzhuang, 5-Shuangcaixiang, 6-Zhenjundian, 7-Xiaopuqiao, 8-Chongrenzhen, 9-Wangjianian, and 10-Chayuan. Red numbers and black numbers represents localities of low-SiO_2_ basalts and high-SiO_2_ alkaline/tholeiitic basalts, respectively. The simplified geologic maps (**a**,**b**) were modified from refs 14 and 39, respectively.

Eastern China hosts widely distributed late Cenozoic basalts (Fig. 1a) that occur in dozens of small-scale flood basaltic fields as well as a few monogenic basaltic fields, have trace element signatures consistent with ocean island basalt (OIB), and are identified as typical intraplate basalts derived from the asthenosphere in a continental setting^7^,^14^. Eastern China therefore represents a prime location for the study of small-scale flood basalts.

**Figure 2:**
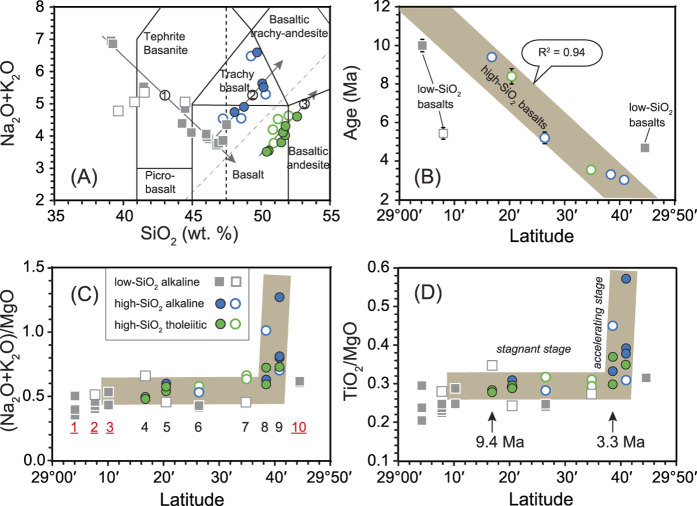
(**A**) Plot of SiO_2_ vs. total alkali. 1, 2, and 3 represent the trends of low-SiO_2_ alkaline basalt, high-SiO_2_ alkaline basalt, and high-SiO_2_ tholeiitic basalt, respectively. (**B–D**): Plots of latitude vs. each of age, (Na_2_O + K_2_O)/MgO, and TiO_2_/MgO, respectively. Ar-Ar and K-Ar ages with errors smaller than symbols are shown without error bars. Closed symbols represent data of the present study, while open symbols represent published data^15^. Sample locations are shown as numbers in (**C**).

In southeast China, four NE-trending Cenozoic volcanic belts are divided according to the distribution of volcanic rocks along major trans-lithospheric faults within the Precambrian Cathaysia Block^15^. Zhejiang province is located at the northeast end of the Cathaysia Block and is cut by three major trans-lithospheric faults (Fig. 1b). Late Cenozoic small-scale flood basalts in this province consist mainly of layered lavas^7^,^16^. Among these flood basalts, the Xinchang–Shengzhou (XS) flood basalt field is the largest, and was emplaced between 10.1 ± 0.3 Ma and 3.0 ± 0.1 Ma (based on nine ages including Ar–Ar and K–Ar dates). XS lavas cover ~500 km^2^ and reach a maximum thickness of ~300 m along the Lishui–Yuyao Fault (Fig. 1b), which divides the Cathaysia Block into western and eastern parts^17^. Aside from a few nephelinites and basanites located at the margin of the field, the XS lavas are mainly olivine basalts and trachybasalts (Fig. 2A). The more alkaline rocks, nephelinites, basanites, and alkali olivine basalts (usually containing mantle xenoliths, and considered to be primary rocks), have ages of ca. 10–5 Ma. Other more evolved lavas (discussed later in the text) show clear negative correlations between Ar–Ar ages (9.4–3.0 Ma) and latitude, displaying a northward-younging eruption trend (Fig. 2B).

## Geochemistry

To understand the distribution of the XS flood basalts, we sampled the basalt field from south to north, and selected 26 fresh samples for geochemical (major and trace elements) and geochronological (K–Ar dating) analyses (Supplementary Tables 1 and 2). These data, as well as published geochemical data and Ar–Ar ages of XS flood basalts^15^, are compiled for discussion. The detailed analytical methods are provided in the Supplementary Methods.

Less silicic samples (SiO_2_ < 47.5 wt.%) display a negative correlation between SiO_2_ and total alkalis, while more evolved samples (SiO_2_ > 47.5 wt.%) deviate from the trends defined by the low-SiO_2_ samples and define two parallel trends that show a positive correlation, one alkaline and one tholeiitic (Fig. 2A, S1). Therefore, the XS basalts can be grouped into low-SiO_2_ alkaline basalts (SiO_2_ < 47.5 wt.%, MgO > 8.5 wt.%), high-SiO_2_ alkaline basalts (SiO_2_ > 47.5 wt.%, MgO < 8.5 wt.%, Na_2_O + K_2_O = 4.5–6.6), and high-SiO_2_ tholeiitic basalts (SiO_2_ > 47.5 wt.%, MgO < 8.5 wt.%, Na_2_O + K_2_O = 3.6–4.6), respectively (Fig. 2A, S1). In addition to lower SiO_2_ contents (39.11–47.38 wt.%), the low-SiO_2_ alkaline basalts are characterized by higher MgO (8.58–11.37 wt.%) and CaO contents (9.73–10.81 wt.%), and higher CaO/Al_2_O_3_ ratios (0.78–1.08) compared with the high-SiO_2_ alkaline/tholeiitic basalts (Fig. S1). Among the high-SiO_2_ alkaline/tholeiitic basalts, samples from the northern XS field which are located to the north of Lishui-Yuyao fault show lower MgO and higher K_2_O and TiO_2_ concentrations than samples from the southern XS field which are located to the south of the fault (Supplementary Table 2).

**Figure 3:**
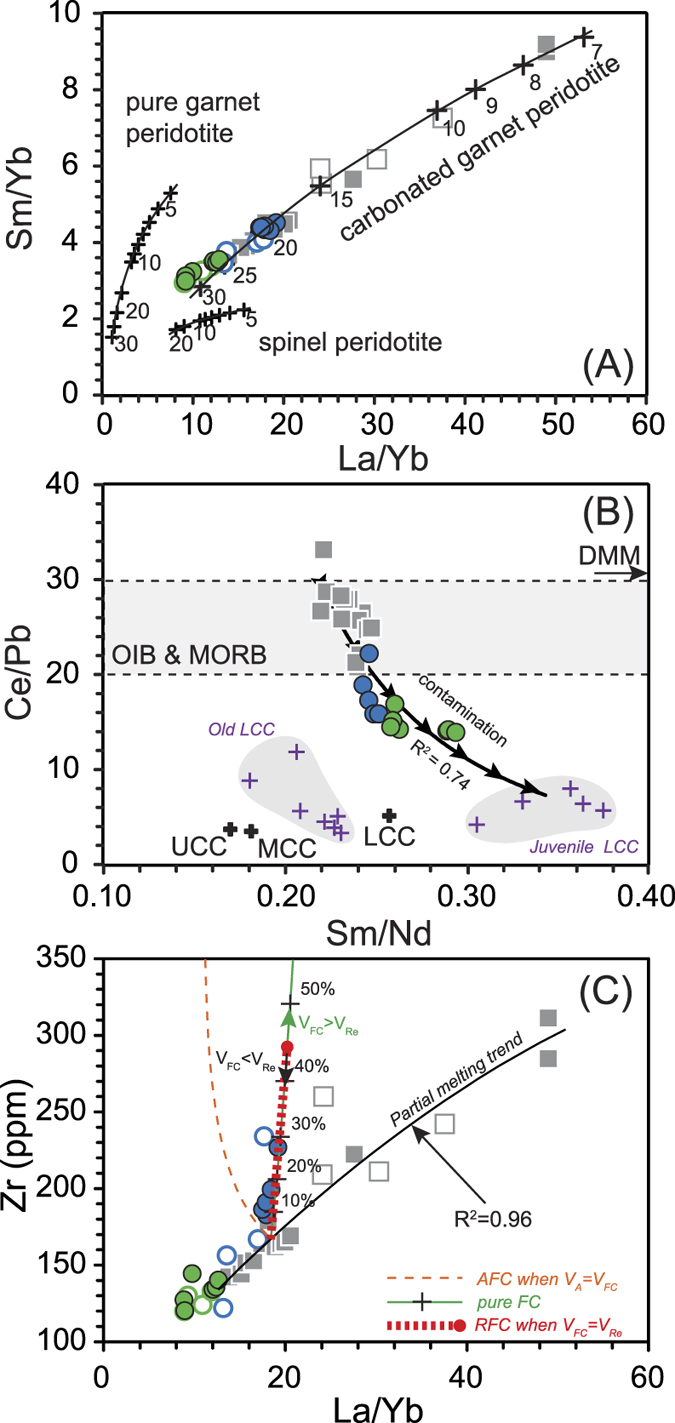
(**A**) Sm/Yb vs. La/Yb. Also shown are batch melting curves calculated for spinel peridotite, garnet peridotite, and carbonated garnet peridotite. The numbers in the plot represent the degrees of partial melting. (**B**) Sm/Nd vs. Ce/Pb. Ranges of typical OIB and MORB Ce/Pb ratios, and DMM composition are after Hofmann^37^ and Workman and Hart^38^. Average upper continental crust (UCC), middle continental crust (MCC), and lower continental crust (LCC) can be referred to Rudnick and Gao^22^. Both old LCC and juvenile LCC are represented by granulite xenoliths from southeast China^26^. (**C**) REAFC modeling results for XS samples plotted as Zr vs. La/Yb. The methods and parameters for modeling are given in the Supplementary Methods. *V*_*A*_, *V*_*FC*_, and *V*_*Re*_ represent the rates of assimilation, fractional crystallization, and recharge during magma chamber processes, respectively.

The chondrite-normalized rare earth element (REE) patterns of the XS basalts are characterized by enrichment in light REEs (Fig. S2) that varies among different rock types (Fig. 3A). In a primitive-mantle-normalized multi-element plot, all samples are characterized by enrichment in highly incompatible elements, showing positive Nb and Ta anomalies, and negative Pb anomalies (Fig. S3). Overall, the degree of enrichment in highly incompatible elements is correlated with lithology; i.e., low-SiO_2_ alkaline basalts are more enriched than high-SiO_2_ alkaline and tholeiitic basalts (La_N_/Yb_N_ for low-SiO_2_ alkaline basalts varys from 9.4 to 33.3; La_N_/Yb_N_ for high-SiO_2_ alkaline/tholeiitic basalts varys from 6.0 to 13.0; N stands for primitive mantle normalised). In addition, the high-SiO_2_ alkaline/tholeiitic basalts show lower Ce/Pb (13.8–22.1), Nb/U (36.4–49.3) and higher Sm/Nd (0.24–0.29) ratios than the low-SiO_2_ alkaline basalts (Ce/Pb = 20.7–33.1; Nb/U = 50.6–57.2; Sm/Nd = 0.22–0.25; Fig. 3B).

## Melting and AFC process

The differences in geochemical trends for the low-SiO_2_ and high-SiO_2_ alkaline/tholeiitic basalts indicate different magma genesis (Figs 2A and 3C). The OIB-like primitive-mantle-normalized trace element patterns (Fig. S3) and high Ce/Pb ratios suggest an asthenospheric source for the low-SiO_2_ alkaline basalts (Fig. 3B). This inference is consistent with the depleted isotopic compositions of low-SiO_2_ basalts in this area (^87^Sr/^86^Sr = 0.70356–0.70368, ε_Nd_ = +4.9 to +5.2^14^,^15^), suggesting a homogeneous asthenospheric source for low-SiO_2_ alkaline basalts. Therefore, the negative correlations between SiO_2_ and incompatible elements such as K, Ti, and Nb in the low-SiO_2_ alkaline basalts are inferred to have been generated by different degrees of partial melting of the mantle source. In addition, the low-SiO_2_ basalts are characterized by high CaO/Al_2_O_3_ ratios (>1); strongly negative Zr, Hf, and Ti anomalies (Hf/Hf* = 0.58–0.60; Ti/Ti* = 0.80–0.82); and super-chondritic Zr/Hf ratios (46.0–48.1), which are common in nephelinites and basanites elsewhere in eastern China^18^,^19^.

These compositional features can be generated by a low degree of melting of a carbonated mantle source^18^. To test this possibility, we use an inverse model to calculate the melting mineral proportions in a garnet peridotite source^20^. Then we can model the batch melting of a hypothetical mantle source in the garnet stability field by La/Yb–Sm/Yb compositions (Fig. 3A; details provided in the Supplementary Methods). Notably, for a given Sm/Yb ratio, all of the XS basalts have higher La/Yb ratios that can be generated solely from a carbonated garnet peridotite source. According to this modeling, the low-SiO_2_ alkaline basalts were generated by a lower degree of melting (5%–15%), while the high-SiO_2_ alkaline/tholeiitic basalts were generated by higher degrees of melting (20%–25%; Fig. 3A), which is consistent with previous calculations^14^.

However, differences in the degree of melting cannot explain the positive correlations between total alkali and TiO_2_ vs. SiO_2_ contents for the high-SiO_2_ alkaline/tholeiitic basalts (Fig. 2A, S1). Positive correlation between MgO and Ni suggests that fractionation of olivine should be common for high-SiO_2_ alkaline/tholeiitic basalts (Fig. S1). Because of the positive correlation between MgO and CaO/Al_2_O_3_, clinopyroxene fractionation should occur and modify the composition of those high-SiO_2_ alkaline/tholeiitic basalts (Fig. S1). Thus elevated contents of incompatible elements (e.g., K, Ti and Zr) in magmas can be generated by fractional crystallization of mafic minerals (e.g., olivine and clinopyroxene; Fig. 3C). Consequently, magma chamber processes should be considered when discussing the genesis of high-SiO_2_ basalts.

**Figure 4:**
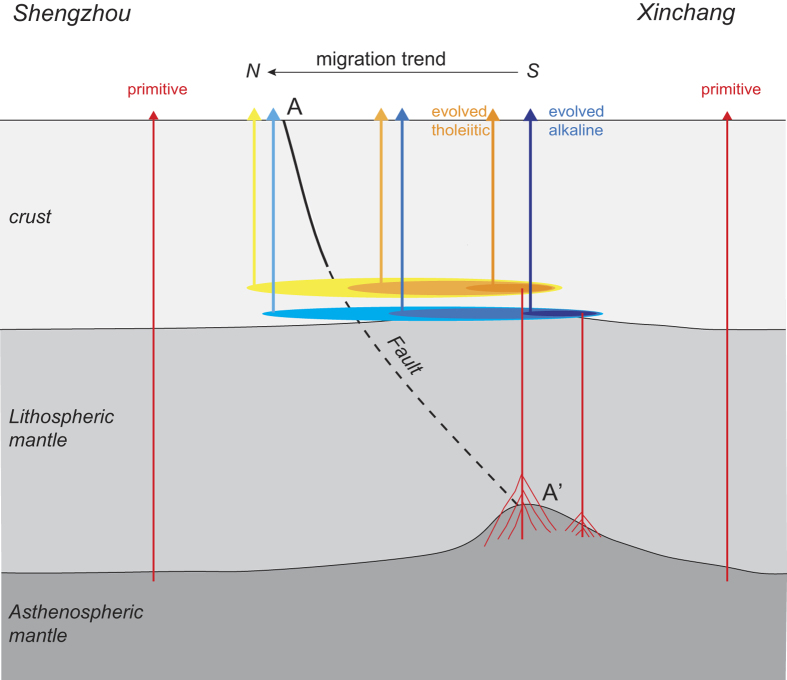
Schematic representation of our model for the genesis of XS basalts. A–A’ represents the Lishui–Yuyao Fault. Primitive alkaline basalts (low-SiO_2_ alkaline basalts) are shown in red, evolved alkaline basalts in blue, and evolved tholeiitic basalts in yellow. Asymmetric magma chamber growth is indicated by the northward extension of the shaded ovals at and just above the lithosphere–crust boundary.

Here, a fractional crystallization model (with or without crustal assimilation) is used to calculate the role of magma chamber processes in the genesis of high-SiO_2_ alkaline basalts^21^ (Fig. 3C). Assimilation of either upper or lower crust would decrease the La/Yb ratio of mantle-derived magma because of the low La/Yb ratio of continental crust^22^. In contrast, pure fractional crystallization of mafic minerals would increase La/Yb (Fig. 3C); the same holds true for other incompatible elements such as Zr, K and Ti (Fig. S1). Due to the young nature and thin crustal thickness of Cathysia terrane^17^,^23^, the garnet effect when lower crust experienced low degrees’ melting should be insignificant. Therefore, the deviation of the high-SiO_2_ alkaline basalts from the low-SiO_2_ alkaline basalt trend in plots of La/Yb versus Zr, and the positive correlation between SiO_2_ and total alkali (or TiO_2_) are controlled by fractional crystallization (Fig. 3C, S1). In addition, crust-sensitive elemental ratios such as Ce/Pb (13.8–22.1), and Nb/U (36.4–49.3), as well as the less depleted Sr and Nd isotopes in the high-SiO_2_ alkaline/tholeiitic basalts (^87^Sr/^86^Sr = 0.70374–0.70424; ε_Nd_ = +1.7 to +5.3^14^,^15^), indicate the significant role of crustal assimilation (e.g. Fig. 3B). Thus, we propose that the geochemical signatures of either high-SiO_2_ alkaline basalts or high-SiO_2_ tholeiitic basalts were controlled not only by the degree of melting of the mantle source, but also by magma chamber processes. Since high-SiO_2_ alkaline/tholeiitic basalts define two independent compositional arrays, an alkaline and a tholeiitic, there must be two independent magma chamber series due to their obvious density difference (Figs 2A and 3C). Formation of low-SiO_2_ alkaline basalts was caused by lower degrees’ melting of homogeneous mantle source which could be depth controlled. When shallow mantle source (Fig. 4) experienced large degrees’ partial melting (>20 wt.%), tholeiitic melts can be formed and show lower density than alkaline basalts^24^. As a result, alkaline basaltic and tholeiitic melts could be trapped in different positions in continental crust due to the fact that locations of basaltic magma chambers are buoyancy determined^24^.

The large density difference between lithospheric mantle and continental crust means that the Moho acts as a mechanical trap for rising melts^25^, frequently, resulting in the formation of mafic magma chambers in the lower crust. However, due to elastic pressurization by replenishment or lowering of the bulk density by fractional crystallization, the magma would eventually rise to the middle or upper continental crust. Each level of continental crust has low Ce/Pb ratios (on average Ce/Pb_UCC_ = 3.7, Ce/Pb_MCC_ = 3.5, and Ce/Pb_LCC_ = 5.0 22; UCC: upper continental crust, MCC: middle continental crust, LCC: lower continental crust), but lower continental crust generally has much higher average Sm/Nd ratios than middle/upper continental crust (on average, Sm/Nd_UCC_ = 0.17, Sm/Nd_MCC_ = 0.18, Sm/Nd_LCC_ = 0.25 22). In addition, evidence from granulite xenoliths suggests that the LCC beneath southeast China is geochemically heterogeneous, and the juvenile LCC is less enriched in LREEs^26^ and has higher Sm/Nd ratios than the old LCC (Fig. 3B). On the plots of Sm/Nd versus Ce/Pb ratios, high-SiO_2_ alkaline/tholeiitic basalts are plotted on the mixing trend to the juvenile LCC with high Sm/Nd ratios. All in all, the lower Ce/Pb and higher Sm/Nd of high-SiO_2_ alkaline/tholeiitic basalts suggest that the magmas were contaminated by lower crust rather than middle/upper crust, and that the magma chambers (both the alkaline and tholeiitic) were most likely located in the lowermost crust (Fig. 3B,C).

## Growth of Magma Chambers

Since fractional crystallization of mafic minerals will decrease MgO and increase total alkali and TiO_2_ concentration in basaltic magma, ratios of (K_2_O + Na_2_O)/MgO and TiO_2_/MgO are sensitive to fractional crystallization and are employed here to assess the degree of magma evolution. Figure 2C,D shows that the degree of evolution of the basalts correlates with their eruptive latitude. High-SiO_2_ alkaline/tholeiitic basalts started to be emplaced in the crust at the southern end of the XS flood basalt field at ca. 9.4 Ma. The composition of the basalts remained roughly unchanged ((K_2_O + Na_2_O)/MgO = 0.40–0.66; TiO_2_/MgO = 0.23–0.35) for ca. 6 Myr (9.4–3.3 Ma). During that time, the flood basalts erupted increasingly to the north over a distance of ~50 km. Once the eruptions reached N29°40’, near the northern end of the XS flood basalt field, the compositional ratios increased rapidly ((K_2_O + Na_2_O)/MgO = 0.60–1.28, TiO_2_/MgO = 0.30–0.57) during 3.3–3.0 Ma, suggesting that evolution of the magmas accelerated toward the end of the magmatic episode (Fig. 2C,D).

Basaltic magma chambers tend to cool rapidly in the crust, and it might not be easy to sustain a magma chamber for 6 Myr. One possible explanation for such an extended residence time is that the basaltic magma chambers experienced continuous replenishment^27^,^28^,^29^,^30^. As discussed above, the low-SiO_2_ alkaline basalts were generated from an asthenospheric source and were erupted after little or no evolution resulting from assimilation and fractional crystallization (AFC). The three ages for the low-SiO_2_ alkaline basalts (10, 5.4, and 4.6 Ma; Supplementary Table 1) young to the north. The oldest low-SiO_2_ alkaline basalt is located at the southern end of the XS basalt field, which indicates the timing of the initiation of eruptions in the area (Fig. 1b). The younger low-SiO_2_ alkaline basalts erupted during the stage with little change in composition (9.4–3.3 Ma; Fig. 2B), suggesting continual melting of the mantle source, and it is probable that fresh primitive melts were injected into the two magma chamber series during that time. Hence, magma replenishment is a plausible explanation for the 6 Myr duration of magma chamber residence in the lower crust.

We use the REAFC (Recharge, Eruption, Assimilation, and Fractional crystallization) model^21^ to test the geochemical variations of the high-SiO_2_alkaline basalts in plots of La/Yb versus Zr (Fig. 3C; details provided in the Supplementary Methods). If the primary basaltic magma experienced AFC (or pure fractional crystallization) processes without magma recharge, it would have become more evolved than the high-SiO_2_ alkaline basalts (Fig. 3C). However, if the basaltic magmas experienced recharge, they would have been buffered against compositional evolution resulting from AFC, and a primitive composition would have been maintained^9^.

Continuous magma recharge would increase and maintain the local temperature at the mantle–crust boundary^27^,^28^. As a result, cooling of the magma would slow, hindering crystallization; such continued replenishment would therefore result in magma chamber growth. In summary, the spatio-temporal distribution of the XS basalts, as well as the presence of regular geochemical variations, indicates replenishment and magma chamber growth in the lower continental crust. However, this raises the question of why the flood basalts migrated northward rather than in other directions.

Here, we consider how local trans-lithospheric faults affect the migration direction of flood basalts. The Lishui–Yuyao Fault, which may represent the suture zone between the western and eastern parts of the Cathaysia block since the Mesozoic^17^,^31^, extends southeastward at depth due to a paleo-subduction event32. Therefore, at the bottom of the lithosphere this fault (A’ in Fig. 4) must be located to the south of its surface expression (A in Fig. 4). The lithospheric mantle around A’ may have been thermo-mechanically eroded by upwelling asthenosphere. In such a case, the degree of melting of the upwelling mantle would have increased and magma chambers would have formed at the crust–mantle boundary, just above A’ and intersecting the southern end of the fault at that depth.

The stress field is geometry-dependent, and stress is concentrated in areas of high curvature such as the margins of magma chambers (in two dimensions^33^). Magma chamber replenishment and growth result in over-pressurization that, if intense enough, would lead to in dike propagation in the direction orthogonal to the least compressive principal stress^28^. The existence of trans-lithosphere fault zones in the north meant that the magma chambers grew asymmetrically in that direction. Therefore, the continuous asymmetrical growth of the magma chambers in the lower crust controlled the northward migration of the XS flood basalts (Fig. 4).

## Conclusions

This study is the first to document the relationship between the temporal-spatial distribution of small-scale flood basalts and the evolution of long-lived basaltic magma chambers, illustrating the importance of magma replenishment in the growth of basaltic magma chambers. We proposed a genetic mechanism that explains how the geochemical compositions of such intraplate basalts are affected by the growth and evolution of magma chambers in the lowermost crust. Furthermore, trans-lithospheric faults can induce asymmetrical magma chamber growth, which is recorded by the spatio-temporal distribution and compositional evolution of small-scale flood basalts. The present results may help to explain the distribution of other flood basalts; e.g., the northwest-southeast-trending distribution of Chifeng flood basalts^8^,^9^, or even the distribution of large igneous provinces; e.g., the north–south-trending distribution of the Columbia River basalts^11^,^36^.

## Additional Information

**How to cite this article**: Yu, X. *et al.* Growing magma chambers control the distribution of small-scale flood basalts. *Sci. Rep.* **5**, 16824; doi: 10.1038/srep16824 (2015).

## Supplementary Material

Supplementary Information

Supplementary Tables
