# Supplementary material for: Growing magma chambers control the distribution of small-scale flood basalts
Source: Sci Rep. 2015 Nov 19;5:16824. doi: 10.1038/srep16824 (PMC4652174; doi:10.1038/srep16824)
Supplement: Supplementary Information [file srep16824-s1.pdf]

Growing magma chambers control the distribution of small-scale flood basalts

Xun Yu, Li-Hui Chen*, and Gang Zeng

State Key Laboratory for Mineral Deposits Research, School of Earth Sciences and Engineering, Nanjing University, Nanjing 210023, China

*Corresponding author: chenlh@nju.edu.cn

Supplementary materials

Methods

1. Major elements and trace elements

The measurements of whole-rock major and trace elements were carried out at the Department of Geology, Northwest University in Xi'an, China. A Rix-2100 X-ray fluorescence spectrometer (XRF) was conducted to measure the major elements. According to the measured values of standards (GSR-1and GSR-3), the uncertainties (relative standard deviation) are about ± 1% for elements with concentrations > 1.0 wt. %, and about ± 10% for the elements with concentrations < 1.0 wt. %. Trace elements were determined with an ELANG100DRC inductively coupled plasma mass spectrometer (ICP-MS) after acid digestion of samples in Teflon bombs. Analyses of the USGS rock standards (BHVO-2, AGV-2, BCR-2 and GSP-1) indicate precision and accuracy better than 5% for Sc, V, Co, Ni, Zn, Ga, Rb, Sr, Y, Zr, Nb, Cs, Ba, U and rare earth elements, and 10% for Cr, Cu, Hf, Ta, Pb and Th. The results for international standards and samples can be seen in Supplementary Table 2 and Table 3.

2. K-Ar dating

Two samples used for K-Ar dating were free from xenoliths and xenocrysts, and were prepared as follows. Each fresh sample was crushed into fragments of 40~60 mesh in size. Phenocrysts like olivine and pyroxene were eliminated by magnetic separation after crushing. Then, 40~60 mg in weight for measurement of argon by isotope dilution, and fragments of an aliquot were ground into powder in an Al_2O_3 Spex mill for the potassium analysis. K abundance (wt. %) were determined using an HG-5 flame-photometer. Isotopic analysis of the extracted argon was measured using the isotope dilution method with a 99.98% pure ^{38}Ar spike on a MM1200 mass spectrometers connected to a purification and extraction system at State Key Laboratory of Earthquake Dynamics, Institute of Geology, China Earthquake Administration, Beijing, China. The decay constants and conversion factors used were those recommended by Steiger and Jager[40]. The results form K-Ar dating work and reported Ar-Ar dating work can be seen in Supplementray Table 1.

3. Inverse and REAFC modeling

(1) Inverse Modeling

The inverse modeling can be referred to Feigenson[20]. Here we make a brief description of the method. REEs show consistent geochemical behavior in normal igneous systems. Thus, by calculating relative changes in REEs patterns of cogenetic basalts, some information about source REEs concentrations, and even mineralogy, can be obtained. Therefore, we can further calculate the melting degrees of the basalts

and know more about the nature of the source to certify our assumptions. As discussed in literature, the only melting mechanism that can be addressed by inverse modeling is equilibrium batch melting. The basic equation for equilibrium melting[41] is:

$$C_l^i = \frac{C_o^i}{D_o^i + F(1-P^i)} \qquad (3\text{-}1\text{-}1)$$

Where C_l^i is the concentration of element *i* in the melt, C_o^i is the concentration of that element in the initial source, D_o^i is the bulk distribution coefficient of *i* in the source, P^i is the sum of partition coefficients of phases in the proportions that they enter the melt, and *F* is the degree of melting. By comparing the REEs to each other, the dependence of concentration on *F* in (3-1-1) can be eliminated. Following Minster and Allegre[42] and Hofmann and Feigenson[43], equation (3-1-1) can be adjusted by normalizing concentration to initial source abundance of the most incompatible REEs:

$$\frac{C^h}{C^i} = S^i C^h + I^i; S^i = \frac{D_o^i}{C_o^i}; I^i = \frac{C_o^h}{C_o^i}(1-P^i) \qquad (3\text{-}1\text{-}2)$$

where C^h refers to the concentration of a highly incompatible element, S^i and I^i are the slope and intercept of the first equation, and can be evaluated from a graph of C^h/C^i versus C^h. This procedure removes the dependence on F and allows comparison of shapes of REEs patterns; however, no explicit information on the absolute degree of melting can be obtained. We use *La* for C^h as Feigenson[20] used in their research work. The detailed steps and plots are not shown here, and readers can refer to Feigenson[20]. After all the steps above, we can get S^i and I^i from linear regressions calculated from C^h/C^i versus C^h combined with various values of input parameter P^i:

$$\frac{C_o^i}{C_o^{La}} = \frac{(1-P^i)}{I^i}; \frac{D_o^i}{C_o^{La}} = \frac{S^i(1-P^i)}{I^i} \qquad (3\text{-}1\text{-}3)$$

A large range of mineral combinations are used to calculated P^i with the only restrictions that P^i must be less than one if all intercepts are positive, and that the shape of P^i must bear some resemblance to D_o^i. This broad range of possibilities translates into the acceptable fields for source concentration and bulk partition coefficients. Therefore, we would use the fit values to further calculate the melting degrees of the source by batch melting equation.

Starting materials for garnet peridotite are ol, 60%; opx, 18%; cpx, 10%; gt, 12%; and starting materials for spinel peridotite are ol, 50%; opx, 15%; cpx, 15%; sp, 20%. Partition coefficients are taken from Johnson[44]. Here we model the melting of spinel peridotite artificially referring to Xu[45], while the melting of garnet peridotite using the inverse modeling (ol, 8%; cpx, 70%; gt, 22% are consumed during melting). The elemental compositions of the start materials and carbonate compositions (about 0.2% is added) are from Workman and Hart[38], and Hoernle[46].

(2) REAFC Modeling

Details about Recharge, Eruption, Assimilation, and Fractional Crystallization (REAFC) model can be referred to Lee[21] and Yu[9]. Here we make a brief description of the method. Following Lee[21], the rate at which the mass M of the magma chamber changes is given by

$$\frac{dM}{dt} = \frac{dM_{re}}{dt} + \frac{dM_e}{dt} + \frac{dM_{cc}}{dt} + \frac{dM_x}{dt} \qquad (3\text{-}2\text{-}1)$$

where the rates of recharge (R), eruption (E), assimilation (A), and fractional crystallization (FC) are dM_{re}/dt, dM_e/dt, dM_{cc}/dt, and dM_x/dt. Eruption and fractional crystallization rates are less than zero because mass is being removed from the magma chamber while recharge and assimilation are greater than zero because mass is being added. Elemental mass balance is then given by

$$dm_{ch} = dM_x C_x + dM_e C_{ch} + dM_{cc} C_{cc} + dM_{re} C_{re} \qquad (3\text{-}2\text{-}2)$$

where dm_{ch} is the change in mass of a given element in the magma chamber. C_{ch}, C_x, C_{cc}, C_{re} are the concentrations of the element in the magma chamber, crystallized minerals, crustal wallrock, and recharging magmas. C_x is related to the concentration in the residual liquid in the magma chamber C_{ch} by an equilibrium partition coefficient D between crystals and melt, $D \equiv C_x/C_{ch}$. After we solved equations above, the evolution lines for magma can be observed. The actual composition of the recharging magma is not known, so we assume that the recharging magma is represented by the average alkaline basalts except for those nephelinites in this study. We also assume that the crystallizing phases are olivine and clinopyroxene with a ratio of ~1. We thus adopt bulk partition coefficients for La, Yb, and Zr as D_{La} ~ 0.017, D_{Yb} ~ 0.175, and D_{Zr} ~ 0.0599 based on Mckenzie and O'Nions[47] and Fujimaki[48]. Compositions of the average lower continental crust is derived from Rudnick and Gao[22]. There are clearly uncertainties with the assumptions we have made. However, because we are interested in general trends, these uncertainties will not affect the first order conclusions.

To facilitate understanding, we use V_{Re}, V_E, V_A, V_{FC} to represent dM_{re}/dt, dM_e/dt, dM_{cc}/dt, and dM_x/dt in the paper. Based on our understanding[9], changing V_E will not change the evolution trend of magmas, thus we only care about the variations of V_{Re}, V_A, V_{FC} during the modeling. When we set V_{Re} is equal to zero, there are only two modes, AFC mode and pure FC mode. In terms of AFC mode, when V_A is larger than V_{FC}, La/Yb ratios of the magmas would be lower (Fig. 3C). Otherwise, the evolution line will get closer to our samples which means that the high-SiO_2 alkaline basalts mainly experienced fractional crystallization with little assimilation. However, no matter AFC mode or FC mode, they cannot explain the stability of the magma for more than six Myr in our observations. So the V_{Re} can not be equal to zero. Here we use RFC mode as an example in Fig. 3C. When the V_{Re} is smaller than V_{FC}, evolution of magma is similar to pure FC mode (green line in Fig. 3C). However, when V_{Re} is much larger than V_{FC}, the evolution of magma will turn back to the primary magma (red line in Fig. 3C).

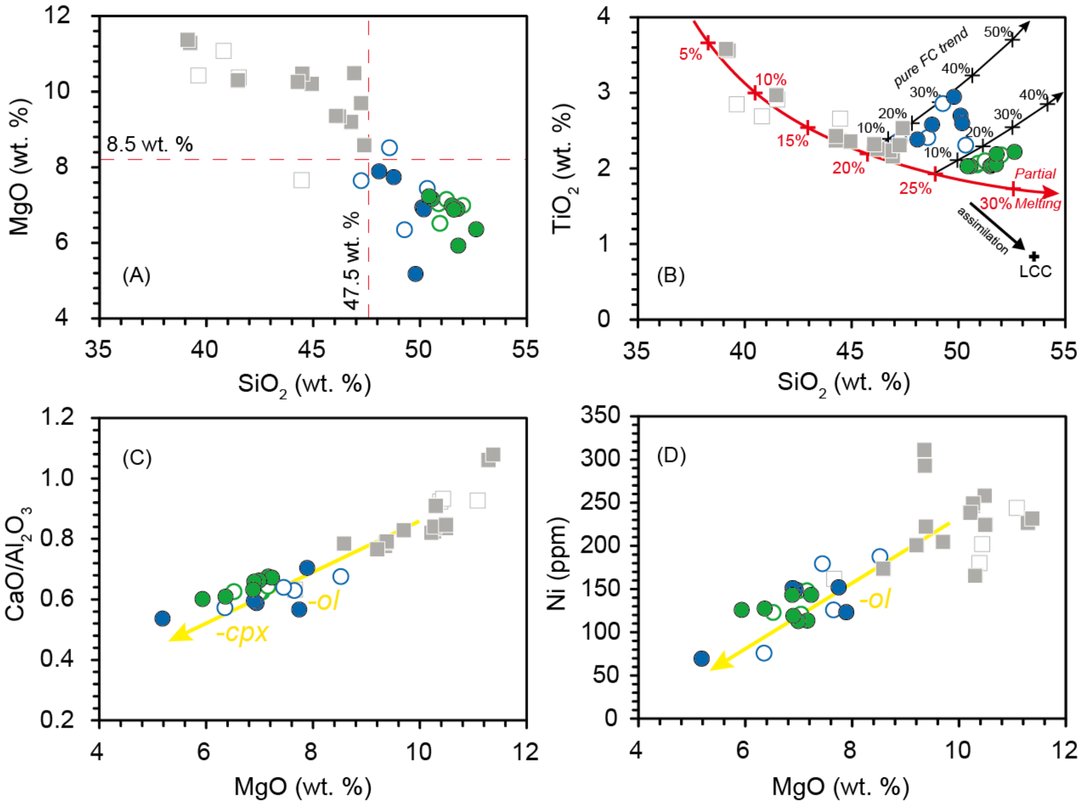

Figure. S1. A, B: Plots of SiO_2 vs. MgO and TiO_2; C, D: Plots of MgO vs. CaO/Al_2O_3 and Ni. In plot B, batch melting, pure fractional crystallization modeling results, and simple mixing trend are showed. Starting melting materials of low-SiO_2 basalts are similar to Fig. 3A. D_{Si} and D_{Ti} for starting materials during melting are based on Johnson[44] and Ayers[49]. During the fractional crystallization we assume D_{Si} ~ 0.8 and D_{Ti} ~ 0.25 which are refer to Johnson[44], Kennedy[50], and Beattie[51]. Average compositions of lower continental crust are from Rudnick and Gao[22].

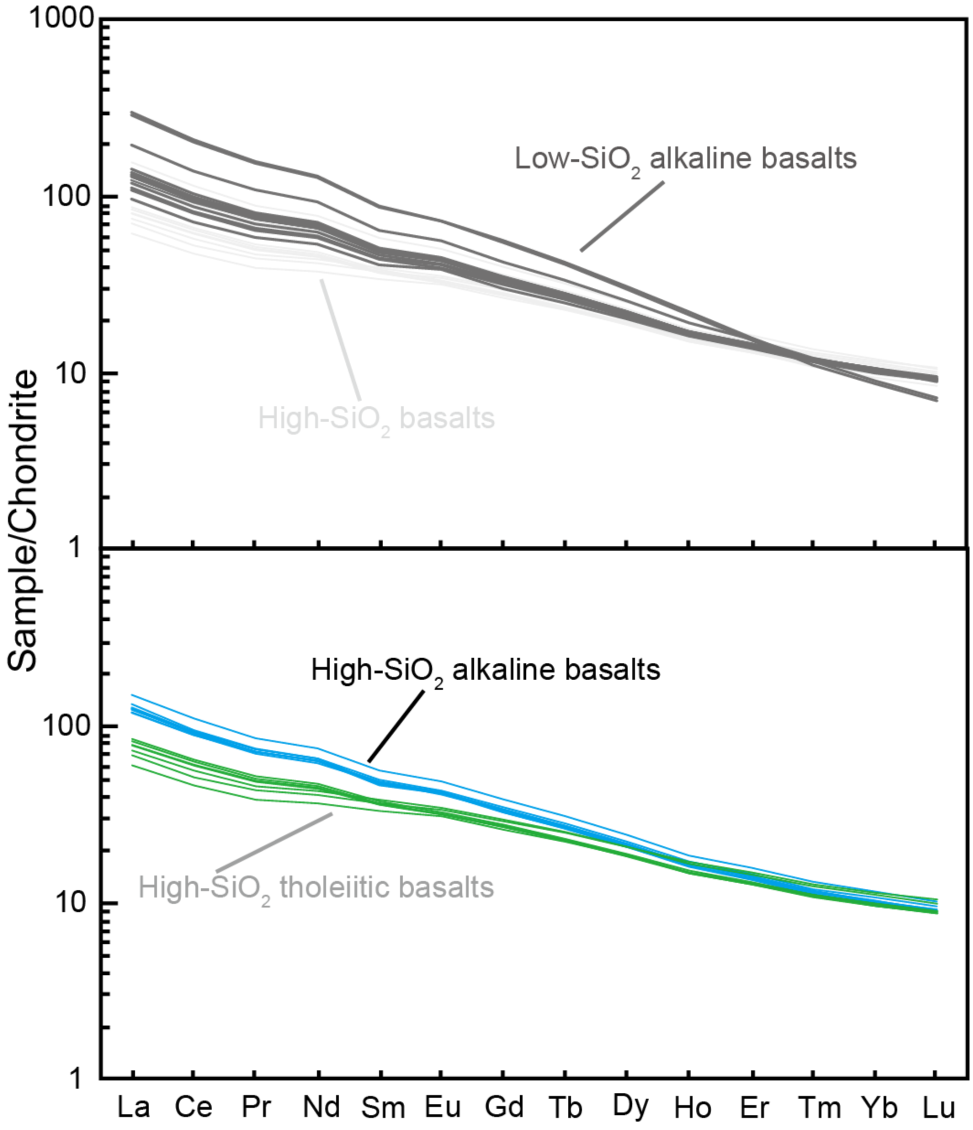

Figure. S2. Chondrite-normalized REE patterns of Cenozoic basalts from Xinchang-Shengzhou flood basaltic field. The chondrite values are from Anders and Grevesse[52].

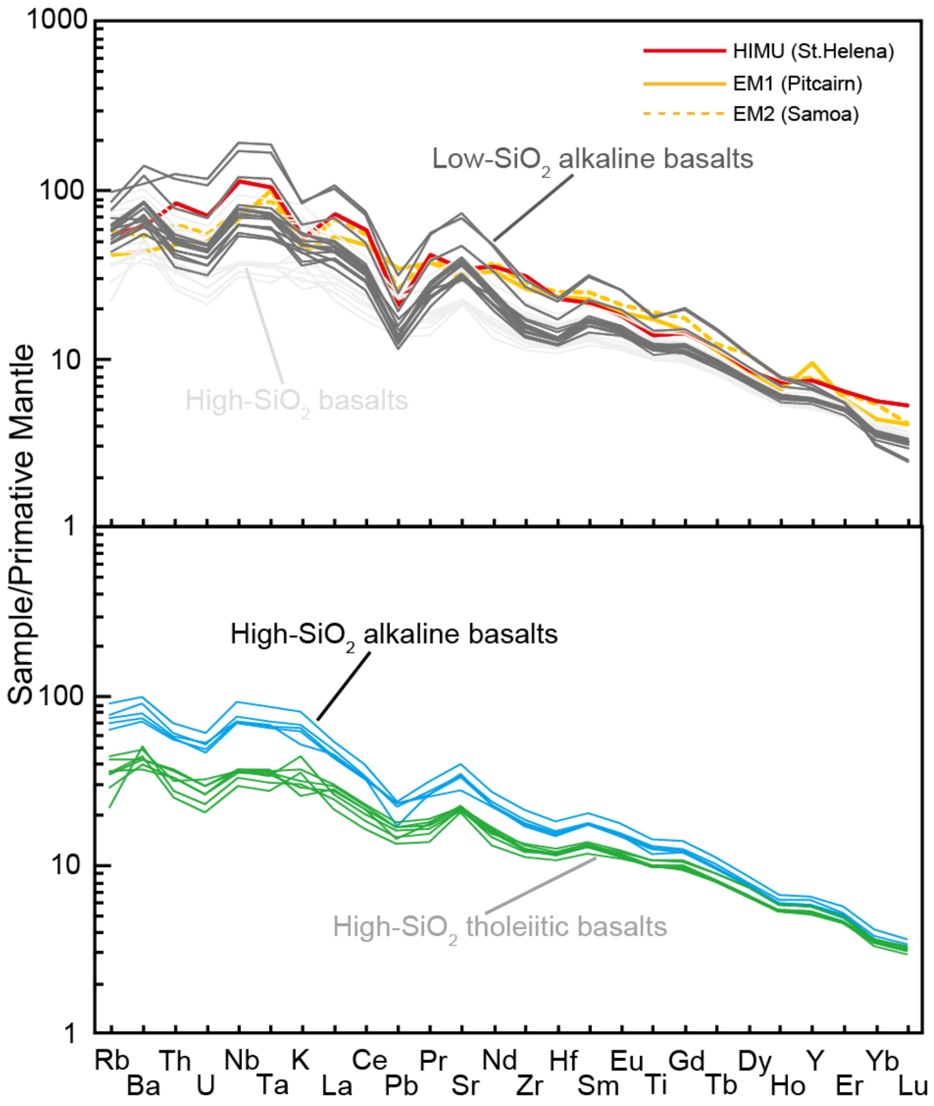

Figure. S3. Primitive-mantle normalized multi-element diagram of Cenozoic basalts from Xinchang-Shengzhou flood basaltic field. The primitive mantle values are from McDonough and Sun[53]. The data of HIMU (St. Helena), EM1 (Pitcairn) and EM2 (Samoa) type basalts are from the geochemical database GEOROC (http://georoc.mpch-mainz.gwdg.de/georoc/). The data used to draw the diagram these basalts are average value of multiple groups of data.

SUPPLEMENTARY REFERENCE

40. Steiger, R. H. & Jäger, E. Subcommission on geochronology: convention on the use of decay constants in geo- and cosmochronology. *Earth Planet. Sci. Lett.* **36**, 359-362 (1977).

41. Shaw, D. M. Trace element fractionation during anatexis. *Geochim. Cosmochim. Acta* **34**, 237-243 (1970).

42. Minster, J. F. & Allègre, C. J. Systematic use of trace elements in igneous processes. *Contrib. Mineral. Petrol.* **68**, 37-52 (1978).

43. Hofmann, A. W. & Feigenson, M. D. Case studies on the origin of basalt. *Contrib. Mineral. Petrol.* **84**, 382-389 (1983).

44. Johnson, K. T. M., Dick, H. J. B. & Shimizu, N. Melting in the oceanic upper mantle: an ion microprobe study of diopsides in abyssal peridotites. *J. Geophys. Res.* **95**, 2661-2678 (1990).

45. Xu, Y.-G., Ma, J.-L., Frey, F. A., Feigenson, M. D. & Liu, J.-F. Role of lithosphere-asthenosphere interaction in the genesis of Quaternary alkali and tholeiitic basalts from Datong, western North China Craton. *Chem. Geol.* **224**, 247-271 (2005).

46. Hoernle, K., Tilton, G., Le Bas, M. J., Duggen, S. & Garbe-Schönberg, D. Geochemistry of oceanic carbonatites compared with continental carbonatites: mantle recycling of oceanic crustal carbonate. *Contrib. Mineral. Petrol.* **142**, 520-542 (2002).

47. McKenzie, D. & O'Nions, R. K. Partial melt distributions from inversion of rare earth element concentrations. *J. Petrol.* **32**, 1021-1091 (1991).

48. Fujimaki, H., Tatsumoto, M. & Aoki, K. -I. Partition coefficients of Hf, Zr, and REE between phenocrysts and groundmasses. *J. Geophys. Res.* **89**, 662-672 (1984).

49. Ayers, J., Dittmer, S. & Layne, G. Partitioning of elements between peridotite and H_2O at

2.0-3.0 Gpa and 900-1100°C, and application to models of subductions zone processes. *Earth Planet. Sci. Lett.* **150**, 381-398 (1997).

50. Kennedy, A.K., Lofgren, G.E. & Wasserburg, G.J. An experimental-study of trace-element partitioning between olivine, ortho-pyroxene and melt in chondrules equilibrium values and kinetic effects. *Earth Planet. Sci. Lett.* **115**, 177-195 (1993).

51. Beattie, P. Systematics and energetics of trace-element partitioning between olivine and silicate melts: Implications for the nature of mineral/melt partitioning. *Chem. Geol.* **117**, 57-71 (1994).

52. Anders, E. & Grevesse, N. Abundances of the elements: Meteoritic and solar. *Geochim. Cosmochim. Acta* **53**, 197-214 (1989).

53. McDonough, W. F. & Sun, S.-S. The composition of the Earth. *Chem. Geol.* **120**, 223-253 (1995).

54. Govindaraju, G. Composition of working values and sample description for 383 geostandards. *Geostandards Newsletters* **18**, 1-158 (1994).
